# Stereotactic body radiotherapy with a single isocentre for multiple pulmonary metastases

**DOI:** 10.1259/bjrcr.20190121

**Published:** 2020-06-18

**Authors:** Ryuji Nakamura, Jun Sugawara, Satoshi Yamaguchi, Hisao Kakuhara, Koyo Kikuchi, Hisanori Ariga

**Affiliations:** 1 Department of Radiology, Iwate Medical University, Morioka, Japan; 2 Department of Radiology, Iwate Prefectural Chubu Hospital, Kitakami, Japan; 3 Department of Radiation Oncology, Iwate Medical University, Morioka, Japan

## Abstract

A 45-year-old male developed a second set of pulmonary metastases 5 years after surgery for extraskeletal mucinous chondrosarcoma of the left shoulder. He already underwent a lobectomy and two segmentectomies for a first set of pulmonary metastases 2 years ago. The closely grouped three nodules within the left lower lung formed a planning target volume (PTV) for stereotactic body radiotherapy (SBRT) with a single isocentre, which was focused on the centre of the largest nodule (the simultaneous plan). Dose-volume histogram analysis confirmed that the plan was superior to an alternative plan, in which SBRT plans would have been produced for each individual tumour (the individual plan). The mean, maximum and minimum PTV doses were 54.0, 57.5 and 47.3 Gy, respectively, in the simultaneous plan, and 65.6, 87.2 and 52.3 Gy, respectively, in the individual plan. The homogeneity index, conformity index, and the maximum dose delivered to the surrounding healthy lung were 1.21, 0.71, and 37.7 Gy, respectively, in the simultaneous plan and 1.66, 4.44, and 46.2 Gy, respectively, in the individual plan. The patient developed Grade two pneumonitis, but remained healthy until 4 years after the SBRT. When multiple closely grouped metastases are treated using SBRT, the use of a single isocentre should be considered.

## Introduction

The concept of oligometastatic disease has led to increased usage of stereotactic ablation radiotherapy (SBRT) as an alternative treatment option for pulmonary metastasis, especially for patients with medically inoperable disease or who are unwilling to undergo surgery.^1–4^ The outcomes of SBRT for pulmonary oligometastasis are comparable to those of surgery, and SBRT is associated with a favourable toxicity profile. In addition, in the era of cancer immunotherapy, SBRT might have advantages over surgery as a treatment for oligometastasis.^5^

Although pulmonary metastasis usually arises as a single lesion, in some cases 3–5 metastatic lesions are treated with SBRT alone or in conjunction with metastasectomy. However, there are limited data about the safety and efficacy of SBRT as a treatment for multiple pulmonary metastases. When multiple closely grouped oligometastatic lesions are treated with SBRT, it is unclear whether a single plan, involving the simultaneous targeting of the lesions using a single isocentre, or multiple SBRT plans, involving individual isocentres for each lesion, should be adopted. In the latter case, the dose contribution cannot be deconvolved for each planning target volume (PTV) separately, and therefore, doses are assessed using a summed plan.^6^ Treating multiple lesions with SBRT becomes more challenging as the distances between the lesions decrease. Therefore, it is necessary to investigate the technical requirements of such treatment.^6^

## Clinical presentation

A 45-year-old male had previously undergone extended radical resection of extraskeletal mucinous chondrosarcoma of the left shoulder 5 years ago. Seven pulmonary nodules, scattered across the bilateral lungs, had been detected 3 years after the operation. In order to remove all of them, partial resection of the left upper and lower lobes and lobectomy of the right lower lobe were performed. The pathological findings of all of the resected nodules were identical to those of the primary shoulder tumour. The patient presented to our department with a second set of pulmonary metastases; that is, three pulmonary nodules within the left lower lobe, 2 years after the first recurrence. The staging procedure, which involved CT and F-18 ﬂuorodeoxyglucose PET, revealed that the primary tumour had not relapsed, and there were no extrapulmonary metastases. SBRT was selected instead of left lower lobectomy, in order to avoid further loss of the patient’s lung parenchyma, as he was still working and asymptomatic and had no significant medical co-morbidities. Pulmonary function tests demonstrated a decreased vital capacity of 2.91 L (80% of the expected value), a forced expiratory volume in 1 s (FEV_1_) of 1.9 L (65% of the expected value), and a FEV_1_/forced vital capacity (FVC) ratio of 0.696, which were indicative of obstructive disease.

**Figure 1.**
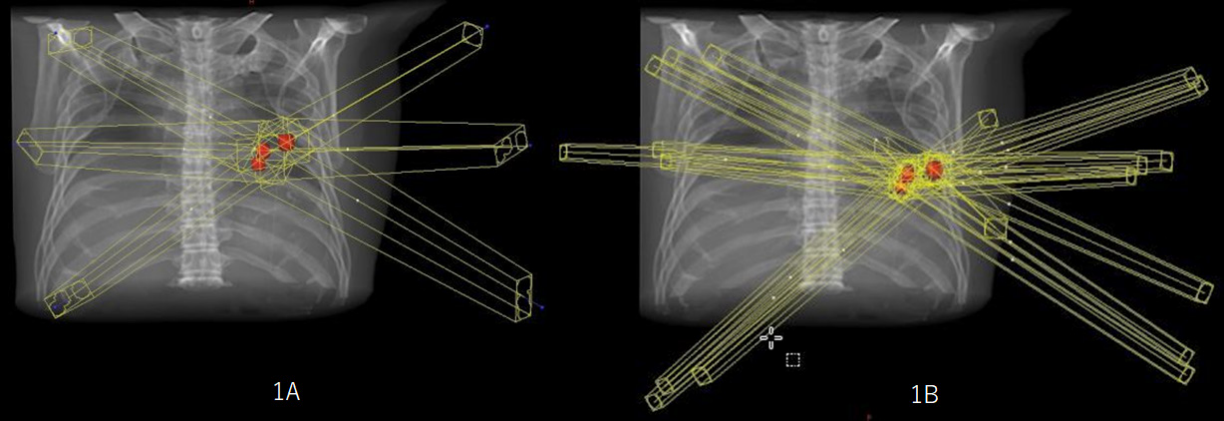
Overview of the SBRT portals used in the simultaneous (1A) and individual (1B) plans

**Figure 2.**
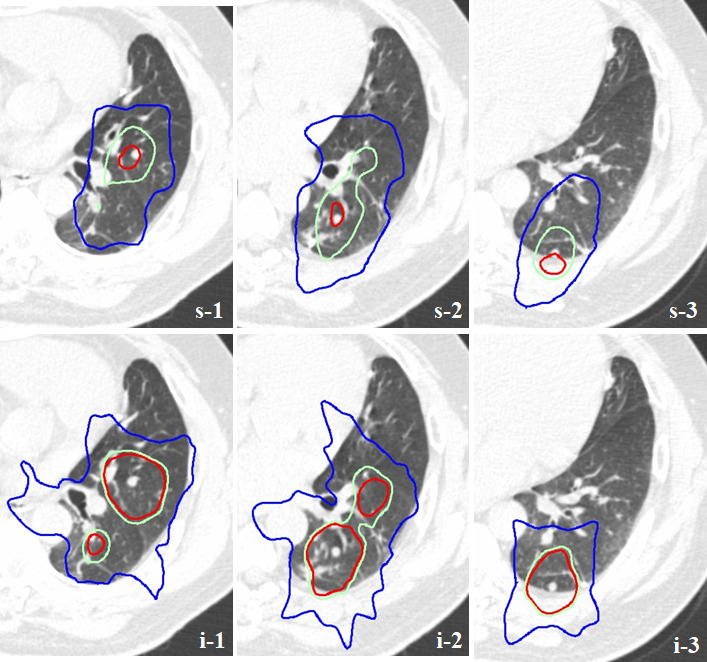
Dose distribution diagram for individual nodules in the simultaneous plan (s-1, s-2, s-3) and the corresponding nodules (i-1, i-2, i-3) in the individual plan The coloured lines represent 105% (red), 95% (green) and 50% (blue) of the prescribed dose levels.

**Figure 3.**
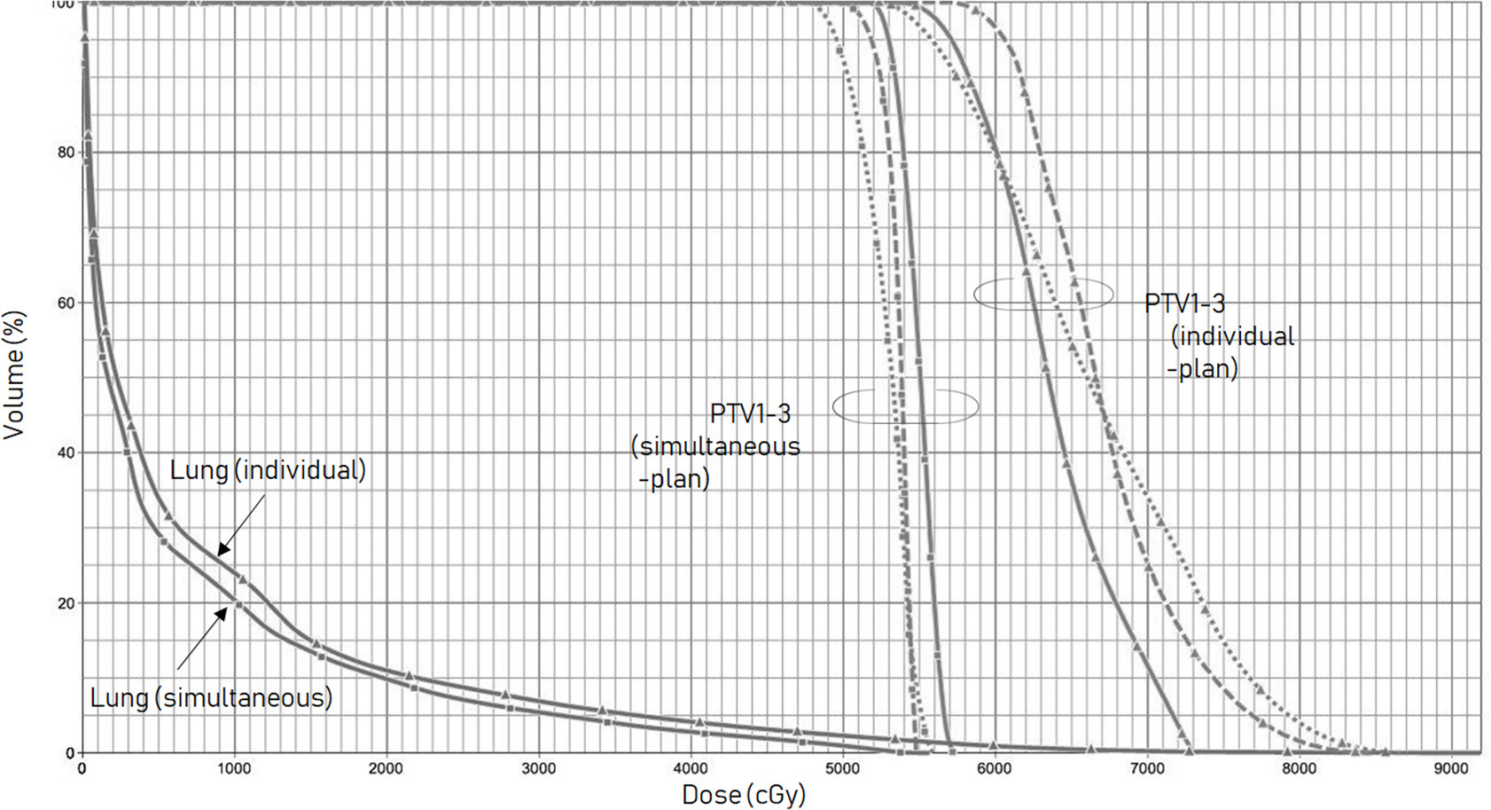
Comparison of the dose-volume histograms for each PTV and the lungs between the simultaneous and individual plans

The patient was immobilized in a body frame (Vac-Lok, Medtec), and a planning CT scan of his chest was performed using a CT simulator (Aquilion; Toshiba, Tokyo, Japan) with a breathing cycle monitoring device (Abches).^7^ The internal target volume was defined as the sum of the volumes of all three tumours on the planning 4D-CT study, and a 5 mm margin was used to generate the PTV in a radiotherapy-planning device (Eclipse V.8.2, Varian Medical Systems, Palo Alto, CA). Cone-beam CT image guidance was used prior to the delivery of each fraction under breath-holding in the deep expiratory phase. The left lung nodules were treated with a 7-field non-coplanar SBRT technique at a dose of 54 Gy, which was delivered in nine fractions over 2 weeks using a linear accelerator (Clinac iX, Varian Medical Systems, Palo Alto, CA). Each port included all three nodules, and multileaf collimators were used to shield the spaces crossing the PTV within the portal area. The isocentre of the fields was localized in the centre of the largest nodule (the simultaneous plan). The angles of the beams and collimators were modified to reduce the portal size as much as possible, while preserving the dose concentration(Figure 1). The composite doses delivered to the organs at risk were calculated to ensure that the relevant dose constraints were adhered to.^8^ A tentative plan, involving the same dose and centre, in which SBRT plans would be created for each of the tumours (the individual plan), was also considered. As a result, it was revealed that the simultaneous plan exhibited a more favourable dose concentration than the individual plan (Figure 2). Dose-volume histogram analysis confirmed that the simultaneous plan was superior to the alternative plan (Figure 3). The mean (D_mean_), maximum(D_max_) and minimum (D_min_) doses delivered to the PTV were 54.0, 57.5 and 47.3 Gy, respectively, in the simultaneous plan and 65.6, 87.2 and 52.3 Gy, respectively, in the individual plan. The homogeneity index ({D_max_-D_min_}/D_mean_), conformity index^9^ and maximum dose delivered to the tissues outside of the PTV plus a 2 cm margin (healthy lung tissue: HLT) were 1.21, 0.71 and 37.7 Gy, respectively, in the simultaneous plan and 1.66, 4.44 and 46.2 Gy, respectively, in the individual plan.

The treatment was well tolerated and did not cause any acute adverse effects. Three months later, the patient developed a cough and shortness of breath. CT showed moderate opacity in the left lower lobe, which gradually disappeared over the next few years. The patient remained healthy until 4 years after the SBRT.

## Discussion

This case involved a rare set of circumstances. The primary tumour was an aggressive and rare soft-tissue sarcoma and was resistant to chemotherapy and radiotherapy.^10^ Although surgery remains the mainstay of treatment for pulmonary metastases, it was deemed unsuitable for our patient, who had a history of extensive surgery, including two partial resections and a lobectomy. SBRT was chosen instead, which is still challenging in cases involving multiple metastases, especially when the lesions are located near to each other. The successful resolution of these difficulties in the present case provides further evidence that SBRT is effective against pulmonary metastases from rare high-grade tumours.

The short distances between targets in the individual plan inevitably would have meant that beams heading to different targets would have overlapped as they reached the targets, which would have resulted in the delivery of excessive doses to the PTV and HLT. The separation of the radiation beams in the simultaneous plan prevented such overlapping at the cost of reductions in the off-isocentre radiation doses delivered to the off-centre targets. However, the off-isocentre dose degradation was negligible due to the short distances between the targets.

Recent studies that attempted to identify HLT dose-based risk factors for radiation-induced pneumonitis after SBRT produced inconclusive results. In the current study, a comparison between the HLT doses of the individual and simultaneous plans revealed a difference in the maximum HLT dose, but not the mean HLT dose, or the V20 dose, which was indicated to be useful for predicting radiation-induced pneumonitis in a previous study.^11^

A mono-isocentric approach to SBRT for multiple brain metastases has recently been developed using intensity-modulated techniques.^12,13^ In the absence of this technique, it is unclear whether the simultaneous method is generally superior to the individual method. It seems that performing SBRT according to the individual plan would have increased the PTV dose without increasing the risk of radiation-induced lung toxicities (RILT). However, the increase in the maximum HPT dose would have been marked, although its relationship with RILT in cases such as ours has not been ascertained. A PTV dose that far exceeds the prescribed dose is not acceptable in radiotherapy. The dose escalation rate is supposed to be non-uniform and to vary according to the distances between nodules or their locations. One of the advantages of the simultaneous treatment method is that it reduces the impact of intrafraction deviation in the positions of the nodules, which is presumed to result in a longer treatment time when repeated SBRT is performed according to the approach adopted in the individual plan.^6^

## Learning points

- SBRT was effective against pulmonary metastases from a radioresistant sarcoma.
- Performing SBRT with a single isocentre is useful for treating multiple closely grouped metastases.
